# Immune-enrichment of insulin in bio-fluids on gold-nanoparticle decorated target plate and in situ detection by MALDI MS

**DOI:** 10.1186/s12014-017-9139-z

**Published:** 2017-01-19

**Authors:** Kai Liang, Hongmei Wu, Yan Li

**Affiliations:** 1 Laboratory of Interdisciplinary Research, Institute of Biophysics, Chinese Academy of Sciences, Beijing, 100101 China; 2 GuangDong Bio-healtech Advanced Co., Ltd, Foshan City, 52800 GuangDong Province China

**Keywords:** Insulin, MALDI MS, Antibody-enrichment, Chemical conjugation

## Abstract

**Background:**

Detection of low-abundance biomarkers using mass spectrometry (MS) is often hampered by non-target molecules in biological fluids. In addition, current procedures for sample preparation increase sample consumption and limit analysis throughput. Here, a simple strategy is proposed to construct an antibody-modified target plate for high-sensitivity MS detection of target markers such as insulin, in biological fluids.

**Methods:**

The target plate was first modified with gold nanoparticle, and then functionalized with corresponding antibody through chemical conjugation. Clinical specimens were incubated onto these antibody-functionalized target plates, and then subjected to matrix assisted laser desorption ionization mass spectrometry analysis.

**Results:**

Insulin in samples was enriched specifically on this functional plate. The detection just required low-volume samples (lower than 5 µL) and simplified handling process (within 40 min). This method exhibited high sensitivity (limit of detection in standard samples, 0.8 nM) and good linear correlation of MS intensity with insulin concentration (R^2^ = 0.994). More importantly, insulin present in real biological fluids such as human serum and cell lysate could be detected directly by using this functional target plate without additional sample preparations.

**Conclusions:**

Our method is easy to manipulate, cost-effective, and with a potential to be applied in the field of clinical biomarker detection.

## Background

The level of insulin in the blood indicates explicitly the function of endocrine beta cells, and thus the metabolism situation of carbohydrates and fat. Thus, insulin is recognized as a marker protein for diagnosis of various types of diabetes and related diseases [1–6]. The detection of insulin in diabetes samples has been used in early-diagnosis, monitoring disease progression, prognosis and pathology research [2]. The progression of technique for insulin analysis will undoubtedly improve the early diagnosis for relative disease and facilitate the follow-up therapy [3, 4].

Currently, insulin analysis is based on a number of methods, including chemiluminescence immunoassays [7], radio immunoassays [8], immune affinity chromatography-LC/MS/MS methods [9], surface plasma resonance immunosensors [10], capillary electrophoretic immunoassay [11], and immune enzymometric assays [12]. However, most of these methods are time- and labor-consuming, of low analysis throughput, and use hazardous reagents such as the radioactive labels.

Matrix assisted laser desorption ionization mass spectrometry (MALDI MS) is a powerful tool for the detection and analysis of biomarkers [13]. Owing to the remarkable features such as high-throughput, high sensitivity, label-free analysis and ease to manipulate, MALDI MS-based analysis has gained considerable interest and is now widely utilized in different fields of biomolecule analysis (including discovery, identification and monitoring), and even as a diagnostic platform [14–17]. Nevertheless, directly detecting low-level biomolecules such as insulin is hampered by the complex composition of real biological fluids. The concentrations of target peptide or protein in blood generally range from nM to μM [18], which were much lower than many other non-target molecules and thus very hard to detect or quantify. In addition, the performance of MS is usually hampered by the high content of salts in biological fluids, known as the effect of ionization suppression [19]. Therefore, several immune-MALDI MS methods have been developed to pre-concentrate target analytes and increase analyzing efficiency. These methods generally applied immune-affinity column [20] or antibody-conjugated magnetic beads [21, 22] to capture target biomolecules, followed by MALDI MS analysis. However, these strategies required additional steps of centrifugation, sample transferring, or column-fractions, which increased sample consumption and limited the analysis throughput [13].

One solution is to specifically enrich target molecules on the MS target plate, to avoid additional handling processes such as column purification or sample transfer. A small number of studies have reported the use of this strategy to detect insulin [23–25]. In these reported methods, the antibody of target analyte was immobilized on 2-dimension planar substrate by using complicated chemical reagents [23, 24] or non-specific adsorption [25]. However, the surface area of 2-dimension planar substrate was restricted, limiting the number of immobilized antibodies and the approach of target analytes [23–25]. Meanwhile, the complex organic chemicals such as dextran [24] may also introduce more non-target signals to MS analysis. Thus, the sensitivities of these strategies were generally in micromole range, hindering a more wide-spread application for the analysis of real biological fluids [23, 25]. Therefore, new design of target plate substrate is still needed to direct analyze real biological fluids using MADLI MS with minimum sample preparation.

Here, to achieve facilitated and sensitive insulin detection, we designed a simple strategy to immobilize insulin antibody directly onto a gold-nanostructured MALDI plate by means of chemical conjugation. The 3-dimension non-planar nano-surface increased surface area, and improved the efficiency of antibody binding, thus enhanced the sensitivity of analysis. Meanwhile, the simple small-molecular chemicals used in this platform avoided introducing interference signals in the MALDI MS. Compared with most previous immune-MALDI MS methods [20–22], this platform could simultaneously enrich and detect target insulin in biological fluids without any off-plate purification by affinity-beads/column, requiring a very small quantity of samples and oversimplified handling process.

## Methods

### Materials

3-Aminopropyltrimethoxysilane (APTMS), 2,5-dihydroxybenzoic acid (DHB), Trifluoroacetic acid (TFA), bovine serum albumin (BSA), human serum albumin (HSA), transferring, immunoglobulin G (IgG), Acetonitrile (ACN), and 1-ethyl-3-(3-dimethylaminopropyl)carbodiimide (EDC) hydrochloride were purchased from Sigma (St. Louis, Mo, USA). 11-mercaptoundecanoic acid (MUA) was obtained from Aldrich (Milwaukee, WI, USA). N-Hydroxysuccinimide (NHS) was purchased from Acros (New Jersey, USA). HAuCl_4_ was purchased from J&K Scientific Ltd. (Beijing, China). NaOH, NaCl, HCl, HNO_3_, absolute ethanol, methanol, ethanolamine (EOA) were of analytical reagent grade from Beijing Chemical Works (Beijing, China). Antibody of human insulin was purchased from the Cell Signaling Technology, Inc. ITO slides (10 Ω/sq) were purchased from Kaivo Electronic Components Co., Ltd. (Zhuhai, China). All reagents and solvents were used as received. Triply distilled water was used for the preparation of all solutions and rinsing.

### Preparation of GNP

The gold nanoparticles (GNP) were prepared according to the previous literature [26]. All glassware used was cleaned in aqua regia solution (HCl:HNO_3_ = 3:1) and then thoroughly rinsed by distilled H_2_O. Briefly, 100 mL of 0.01% HAuCl_4_ was heated to boiling in a round-bottom flask equipped with a condenser. Then 1.3 mL aqueous solution of sodium-citrate (1%) was added under vigorous stirring. In about 25 s, the solution turned blue; in approximately 1 min, the blue color change to red-violet gradually. After that the solution was kept boiling for an additional 10 min, and then cooled at room temperature. The prepared nanoparticle was stored at 4 °C.

### Surface modification of ITO slide

#### Silylation of ITO slide

The ITO glasses were cut into 25 × 37.5 mm slides and cleaned by sequential sonication in acetone for 20 min, in isopropanol for 20 min, in soap water for 15 min, and twice in distilled water for 10 min. The cleaned ITO slides were immersed in 5 M NaOH for 8 h at room temperature and then flushed thoroughly with distilled water and dried under N_2_ stream. Afterwards they were immediately treated in a freshly prepared APTMS solution (3% in methanol) for 2 h. The resulting APTMS-ITO slides were sonically cleaned in methanol for three times and dried under N_2_ stream.

#### Deposition of GNP on APTMS-ITO

The APTMS-ITO slides were immersed in GNP solution for at least 8 h at 4 °C prior to flushing with distilled water and dried under N_2_ stream.

#### Derivatization of carboxyl group on GNP-ITO slide

The GNP deposited ITO (GNP-ITO) slides were immersed in a solution of MUA in ethanol (10 mM) for 8 h, rinsed with ethanol, and then dried under N_2_ stream.

#### Immobilization of protein on MUA-GNP-ITO

To covalently attach protein on these two kinds of substrate, an aqueous solution of EDC (75 mM) and NHS (15 mM) was first applied to treat the slides for 25 min at room temperature. Then the protein solutions (anti-insulin or BSA in pH 7.4 phosphate buffer) were spotted directly on the proper locations of the slides using pipettor. After all samples were spotted, the slides were laid in a sealed humid bottle at room temperature for at least 2 h to complete coupling reaction. Then an aqueous solution of EOA (1 M, adjusted with 5 M HCl to pH 8.6) was applied to treat the slides for 1.5 h to block the unreacted carboxyl groups. Finally, the anti-insulin or BSA modified ITO slides were flushed thoroughly with water to clean the unbound proteins and dried under N_2_ stream.

#### Insulin immune-reaction and MALDI-TOF analysis

Human insulin (0–72 nM) was dissolved in pH 7.4 phosphate buffer (10 mM) or PBS buffer containing albumin (35 mg/mL), transferring (2 mg/mL) and IgG (6 mg/mL) to generate standard insulin solutions. Five microliter of insulin solution was dropped on the anti-insulin modified ITO slide and incubated on a shaking table at room temperature for 30 min. Then the ITO plate was rinsed with Tween 20 solution (0.05% Tween 20 in water) and distilled water sequentially and dried under N_2_ stream. After that, 1 μL of DHB (15 mg/mL, 50% ACN, 0.1% TFA) was applied as the matrix for MALDI-TOF mass analysis. The solvent was dried naturally under room temperature and then the target plate was subjected to MALDI MS analysis.

Mass spectrometry was acquired on SHIMADZU AXIMA Resonance MALDI-IT-TOF on reflective/positive ion mode. Laser power of 105 mV was selected as standard desorption energy in MS analysis.

## Results and discussion

### Strategy to anchor insulin antibody on MALDI MS target plate

An indium tin oxide coated glass slide (ITO slide) was selected as MALDI MS target plate due to its ease of chemical modification and excellent conductivity [26], the latter is important for the efficient MALDI MS analysis [25]. An easy approach to construct nano-surface was designed, and used to immobilize the insulin antibody on the ITO slide by chemical conjugation.

**Fig. 1:**
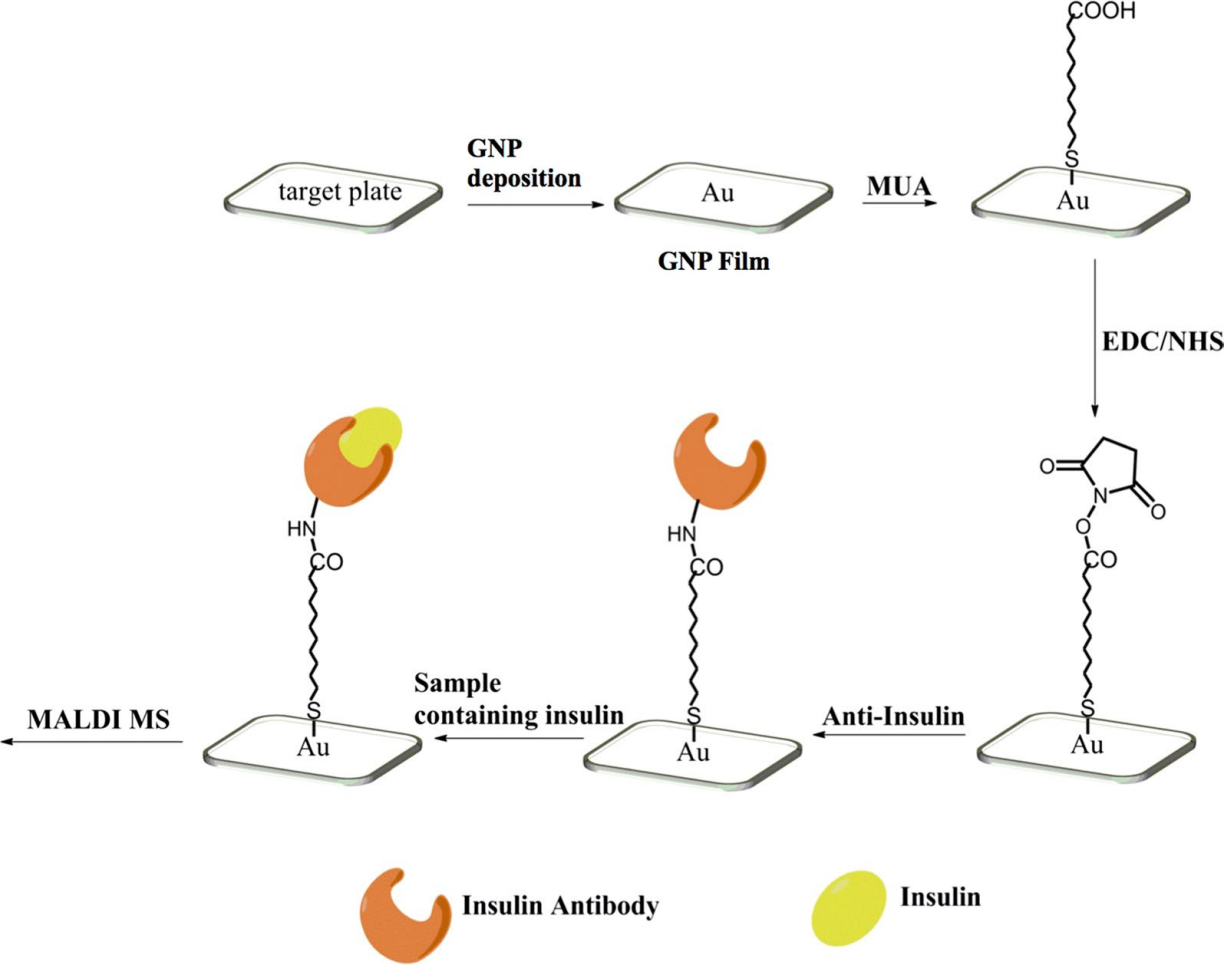
Schematic overview for the preparation of GNP-modified target plate and antibody-assisted enrichment and MS-detection of insulin

**Fig. 2:**
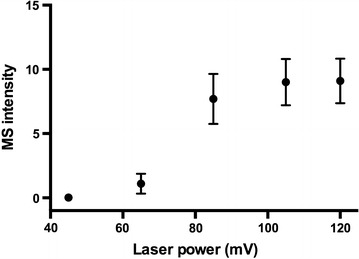
MALDI MS intensity of standard insulin sample (8 nM) obtained at different laser energy power (45, 65, 85, 105, and 120 mV). Data represent MEAN ± SEM, n ≥ 3

The approach (Fig. 1) utilized gold-nanoparticle (GNP) to modify ITO surface. Three key steps were needed: derivatization of amino group on ITO slide with silanization reagent APTMS [27]; deposition of GNP on the amino-terminated ITO slides [26]; derivatization of carboxyl group on the GNP-ITO and chemical coupling of antibody. The GNP with negative charged surface could be adsorbed stably on the amino-terminated ITO surface through electrostatic interactions [26]. Compared with the 2-dimension planar surface of original ITO, the 3-dimension nano-structure would greatly increase the surface area of target plate. Correspondingly, the antibodies immobilized on plate surface increased, which was beneficial for capturing more insulin and increasing MS sensitivity [6]. A key factor to affect detection was the laser energy of MALDI MS, because weak laser power may not desorb the insulin immobilized by antibody. As shown in Fig. 2, when the laser energy was lower than 65 mV, the signal was hardly detected. The MS signal intensity increased gradually as laser energy rose from 65 to 105 mV, and kept stable after laser energy exceeded 105 mV (Fig. 2). Because excessive laser energy may introduce additional noisy signal, we chose 105 mV as standard laser energy in our experiments to achieve the best signal intensity.

**Fig. 3:**
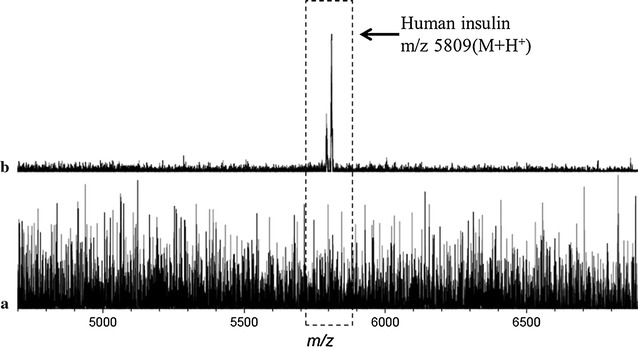
MALDI MS spectra of standard insulin samples (8 nM). **a** MS Spectrum achieved on BSA modified GNP-ITO without anti-insulin antibody. **b** MS Spectrum achieved on anti-insulin antibody-modified GNP-ITO. 5 Replicates for each data were tested at the same conditions

To prove the validity of this approach, either insulin antibody or a control protein BSA were covalently anchored on the GNP-ITO slides. Subsequently, standard solutions of insulin were incubated on these two protein-modified plates, and then washed to remove any unbound or weakly bound species. As shown in Fig. 3a, on the BSA modified GNP-ITO (without antibody), no insulin signal could be detected (Fig. 3a). Meanwhile, by using the antibody-modified GNP-ITO as target plate, clear signal of insulin was identified (Fig. 3b), indicating that the insulin in samples was captured on the antibody-functionalized plate and then detected by MALDI MS. Notably, there is no other noisy signals visible in the mass range, indicating that the designed target plate did not introduce interfering signals in this range.

**Fig. 4:**
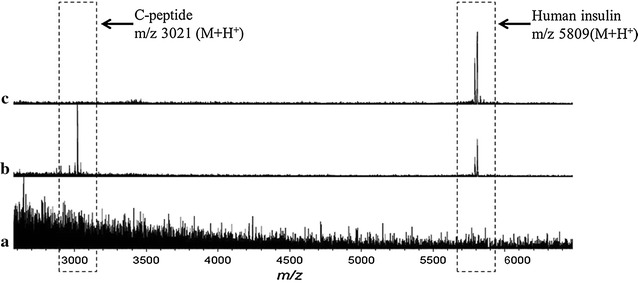
MALDI MS spectra of standard insulin (34 nM)/C-peptide (331 nM) mixture solution. **a** MS Spectrum achieved on BSA modified GNP-ITO without anti-insulin antibody. **b** MS Spectrum achieved on conventional target plate. **c** MS Spectrum achieved on anti-insulin antibody-modifed GNP-ITO. 5 Replicates for each data were tested at the same conditions

To exclude the possibility of capturing non-target molecules on the antibody-modified GNP-ITO, a mixture solution of insulin (34 nM) and non-target peptide (331 nM) was analyzed by using different target plates. As shown in Fig. 4, no recognizable peak can be observed on the BSA modified GNP-ITO (Fig. 4a). In addition, the conventional MALDI MS plate demonstrated abundance of both two peptides (Fig. 4b). As no separation method employed, the insulin signal was much lower than C-peptide (Fig. 4b). In contrast, on the antibody-modified target plate, the signal of C-peptide disappeared and only the clear peak of insulin was observed in spectrum (Fig. 4c). These evidences revealed that the antibody have been immobilized substantially on the target plate and specifically capture the target insulin in samples as we expected.

### Sensitivity of the MALDI MS based on the anti-insulin modified GNP-ITO

**Fig. 5:**
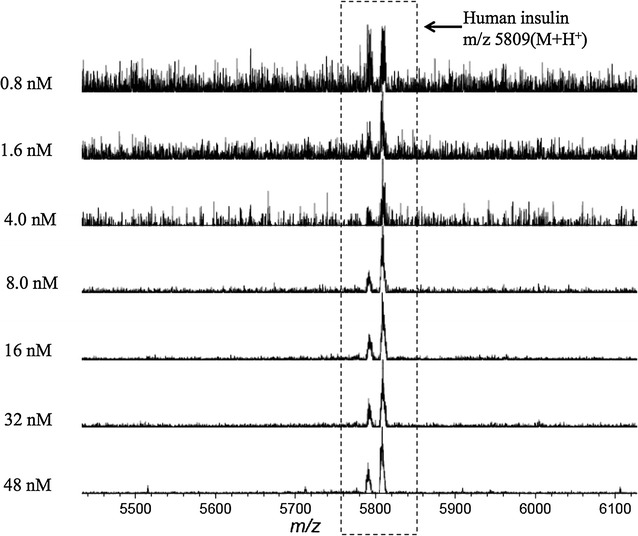
MALDI MS spectra of standard insulin samples in different concentrations (0.8, 1.6, 4, 8, 16, 32, 48 nM) achieved on anti-insulin antibody-modified GNP-ITO. 3 Replicates for each data were tested at the same conditions

**Fig. 6:**
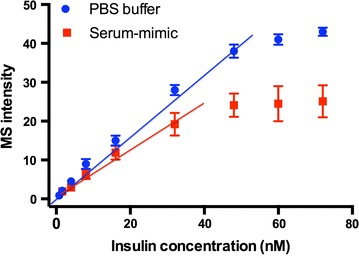
Correlation of MS intensity with the concentrations of insulin (0.8, 1.6, 4, 8, 16, 32, 48, 60, and 72 nM) spiked in PBS buffer (*blue circle*) or artificial serum-mimic (*red square*) containing albumin (35 mg/mL), transferring (2 mg/mL) and IgG (6 mg/mL). Data represent MEAN ± SEM. n ≥ 3

Standard insulin samples at different concentrations were tested on the antibody-modified GNP-ITO. Five microliters of insulin solution were incubated on a circular area (diameter 4 mm) of the target plate for 30 min followed by washing with Tween 20 solution (0.05%) and water. After drying, DHB (15 mg/mL in 50% ACN, 0.1% TFA) was spotted on the circular area as MS matrix and the target plate was subjected to MALDI MS analysis. As illustrated in Fig. 5, strong signals of insulin at m/z 5809 and 5791 were clearly detected. The insulin peaks could be resolved from the background (≥3 times of baseline intensity) even when the insulin level was as low as 0.8 nM. The limit of detection (LOD) at 0.8 nM obtained in standard solution is much lower than the previous reported similar works, in which the tested peptide levels were usually at the range of micromole [25] or several hundred nano-moles [24]. A good linear correlation (R^2^ = 0.994) of MS intensity with insulin level was obtained in the range of 0.8–48 nM (Fig. 6). We also tried to test the performance of our method in complex bio-fluids. Due to the difficulty to obtain insulin-free serum, we tried to mimic the serum by generating a buffer solution containing serum-abundant proteins such as albumin (35 mg/mL), transferring (2 mg/mL) and IgG (6 mg/mL), which were added at the concentrations similar to real serum. As shown in Fig. 6, although the MS intensity of insulin based on artificial matrix was lower than PBS buffer, a linear relation with R^2^ = 0.985 still could be observed in the range lower than 32 nM (Fig. 6). These results demonstrated the ability of our method to perform real bio-fluid analysis, and that the interference from sample matrix was limited.

A serial of parallel serum samples collected from 6 patients accepting insulin therapy were also tested to investigate the reproducibility. Good repeatability was obtained in within and between day tests, as shown in Table 1. In short, sensitive detection of insulin was easily achieved on this developed target plate, just requiring very low-volume sample and oversimplified handling process.

**Table 1:** Repeatability of MALDI MS results obtained on antibody-modified GNP-ITO in 6 patient serum samples (accepting insulin therapy)

	Mear (MS intensity)	CV% for within day	CV% for between day
Patient 1	2.50	15.8	17.0
Patient 2	4.52	13.8	14.8
Patient 3	4.11	14.1	15.3
Patient 4	3.28	15.0	15.3
Patient 5	4.47	11.1	14.6
Patient 6	3.67	15.1	16.0

Ten replicate measurements per sample per day were performed for 3 days to generate 30 values per sample

### Analysis of biological fluids

**Fig. 7:**
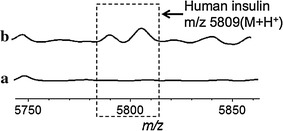
MALDI MS spectra of human serum samples. *a* MS Spectrum achieved on conventional target plate. *b* MS Spectrum achieved on anti-insulin antibody decorated GNP-ITO. 3 Replicates for each data were tested at the same conditions

**Fig. 8:**
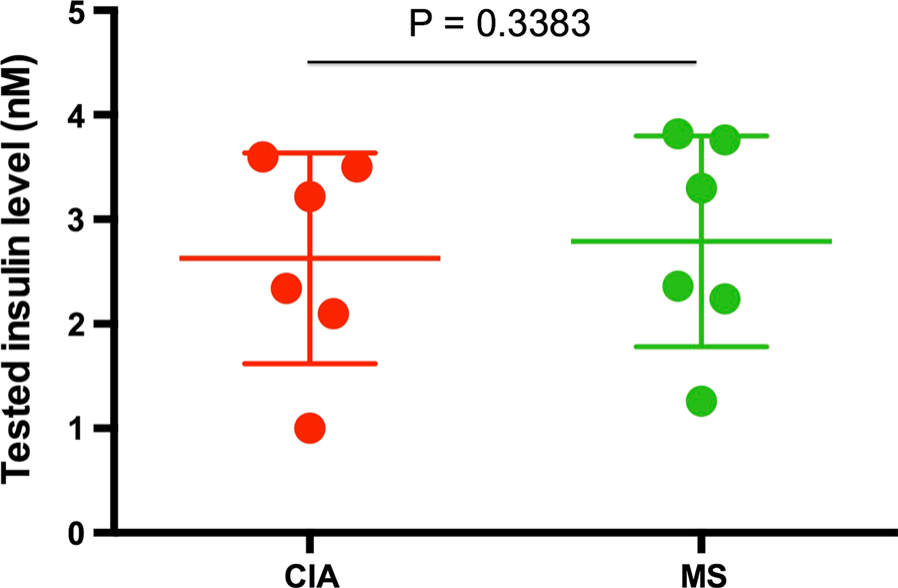
Comparison of serum insulin levels tested by chemiluminescence immunoassays (CIA) and the MALDI MS based on antibody-modified GNP-ITO (MS). Differences with *p* values <0.05 were considered statistically significant. Data represent MEAN ± SEM

It is difficult to directly detect low-level targets in complex samples using MALDI MS. The complicated composition and high level non-target molecules in biological fluids generally generated high background signals and interfered with the detection of target molecules. In addition, salts present in samples will greatly reduce the performance of MS analysis. Figure 7a indicated the MS spectrum by applying raw human serum (from the patients accepting insulin therapy) on the conventional MALDI plate, and no identified signals can be observed at m/z 5809 and 5791. Subsequently, the human sera were incubated and washed on the antibody-modified GNP-ITO and then subjected to MALDI MS analysis. As shown in Fig. 7b, the signals of insulin at m/z 5809 and 5791 were discerned clearly from the background, representing a significant improvement compared with conventional MALDI MS methods. According to the MS intensity, the concentrations of insulin in these serum samples were calculated as shown in Table 2. Compared to the insulin level obtained by clinically standard chemiluminescence immunoassays (CIA), the immune-MS results did not show significant difference, with a *p* value >0.05 (*p* = 0.3383, Table 2; Fig. 8). Meanwhile, strong correlation of the results tested by CIA with immune-MALDI MS was also observed (correlation coefficient >90%).

**Table 2:** Insulin levels in 6 human serums tested by chemiluminescence immunoassays (CIA) and the developed immune-MS method

	Serums of patients (accepting insulin therapy)					
	#1	#2	#3	#4	#5	#6
CIA (nM)^a^	1.0	3.6	3.2	2.1	3.5	2.3
MS (nM)^b^	1.3	3.8	3.3	2.2	3.8	2.4

5 Duplicates of each sample were tested by MALDI MS based on antibody-modified target plate and CIA

^a^ *CIA* chemiluminescence ImmunoAssays

^b^ *MS* MALDI MS based on antibody-modified target plate

**Fig. 9:**
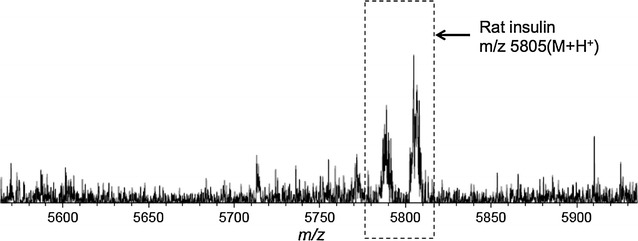
MALDI MS spectrum of cell lysate in rat pancreas achieved on anti-insulin antibody-modified GNP-ITO. 3 Replicates for each data were tested at the same conditions

In addition, we measured the lysate fluid of rat pancreas cell using this developed target plate. The amino acid sequences of rat insulin ([M + H]^+^=5805 Da) and human insulin ([M + H]^+^=5809 Da) although differ by several amino acids, the rat insulin could still be recognized by the anti-insulin we applied. The MS spectrum (Fig. 9) revealed that the rat insulin in lysate fluid also could be directly detected, with a concentration calculated of around 14 nM. These results further exhibited the feasibility of this method for bio-fluids analysis.

## Conclusion

Here, we proposed a strategy to construct antibody-functionalized MALDI target plate for fast and high-throughput MS analysis of insulin. The nanostructure on the surface of target plate improved the efficiency of antibody coupling and insulin detection. Biological fluids, such as serum or cell lysate, could be sensitively analyzed using this functional target plate without any pre-purification, which is impossible for the conventional MALDI MS methods. Furthermore, our method is also useful for the immobilization of other antibodies and analysis of other types of biomolecules, thus offering a widely-used toolbox for the fields of bio-sample exploration.

## Abbreviations

APTMS: 3-aminopropyltrimethoxysilane
DHB: 2,5-dihydroxybenzoic acid
TFA: trifluoroacetic acid
BSA: albumin bovine serum
ACN: acetonitrile
EDC: 1-ethyl-3-(3-dimethylaminopropyl)carbodiimide
MUA: 11-mercaptoundecanoic acid
NHS: *N*-hydroxysuccinimide
EOA: ethanolamine
ITO: indium tin oxide
GNP: gold nanoparticles
MALDI MS: matrix assisted laser desorption ionization mass spectrometry
